# Very Long-Term (15 to 23 Years) Outcomes of Successful Balloon Angioplasty Compared With Bare Metal Coronary Stenting

**DOI:** 10.1161/JAHA.112.004085

**Published:** 2012-10-25

**Authors:** Kyohei Yamaji, Takeshi Kimura, Takeshi Morimoto, Yoshihisa Nakagawa, Katsumi Inoue, Shoichi Kuramitsu, Yoshimitsu Soga, Takeshi Arita, Shinichi Shirai, Kenji Ando, Katsuhiro Kondo, Koyu Sakai, Masashi Iwabuchi, Hiroyoshi Yokoi, Hideyuki Nosaka, Masakiyo Nobuyoshi

**Affiliations:** Division of Cardiology, Kokura Memorial Hospital, Kitakyushu, Japan (K.Y., S.K., Y.S., T.A., S.S., K.A., K.K., K.S., M.I., H.Y., H.N., M.N.); Department of Cardiovascular Medicine, Kyoto University Graduate School of Medicine, Kyoto, Japan (K.I.); Center for General Internal Medicine and Emergency Care, Kinki University School of Medicine, Osaka, Japan (T.K.); Division of Cardiology, Tenri Hospital, Japan (T.M.); Division of Laboratory Medicine, Kokura Memorial Hospital, Kitakyushu, Japan (Y.N.)

**Keywords:** bare metal stent, coronary artery disease, late restenosis, percutaneous transluminal coronary angioplasty, prognosis

## Abstract

**Background:**

Target lesion revascularization (TLR) continues to occur beyond 4 years after bare metal stent (BMS) implantation. However, long-term outcomes after balloon angioplasty (BA) compared with BMS are currently unknown.

**Methods and Results:**

From 1989 to 1990, 659 patients (748 lesions) underwent successful BA with final balloon ≥3.0 mm excluding patients with acute myocardial infarction and were compared with 405 patients (424 lesions) with BMS implantation from June 1990 to 1993. Cumulative incidences of death and target lesion thrombosis (>1 year) were similar between the BA group and the BMS group (44.4% versus 45.4%, *P*=0.60; and 1.5% versus 0.7%, *P*=0.99; respectively). Cumulative incidence of TLR during overall follow-up was significantly higher after BA than after BMS implantation (44.6% versus 36.0%, *P*<0.001), whereas cumulative incidence of late TLR (>4 years) tended to be lower in the BA group than in the BMS group (16.3% versus 21.4%, *P*=0.16). Cumulative incidence of late TLR after BA was significantly lower in patients with small percent diameter stenosis (%DS) at early follow-up angiography compared with large %DS (14.5% versus 28.0%, *P*=0.02). In lesions with serial angiography, late lumen loss from early (6 to 14 months) to long-term (4 to 10 years) follow-up angiography was significantly smaller in the BA group (n=42) than in the BMS group (n=55) (−0.08±0.45 mm versus 0.11±0.46 mm, *P*=0.047).

**Conclusions:**

Compared with BMS implantation, BA was associated with a trend for less late TLR beyond 4 years and with significantly smaller late lumen loss from early to long-term follow-up angiography.

## Introduction

Coronary artery stents have been widely used since the 2 landmark trials (the Stent Restenosis Study [STRESS] and the BElgian NEtherlands STENT I [Benestent-I] study) demonstrated the efficacy of bare metal stents (BMSs) in reducing restenosis and repeated revascularization compared with balloon angioplasty (BA).^1,2^ The midterm follow-up study showed continued benefit of coronary stenting in rate of target lesion revascularization (TLR).^3^ However, long-term studies comparing balloon angioplasty and coronary artery stenting have currently been very limited.

We previously reported that late TLR (beyond 4 years after BMS implantation) had not occurred infrequently (1.6% per year) after an apparent plateau phase between 1 and 4 years.^4,5^ Furthermore, from serial angiographic analysis, late luminal renarrowing at the site of BMS implantation seemed to be a progressive process starting from 3 to 4 years and extending beyond 10 years. Human postmortem histopathologic studies evaluated beyond 3 to 5 years after stent placement have suggested that this progressive luminal narrowing and the corresponding late TLR might be related to chronic inflammatory reactions and in-stent neoatherosclerosis in response to the permanent presence of metal devices.^6–8^ Use of polymer-based metallic drug-eluting stenting (DES) has been demonstrated to be associated with marked reduction of early restenosis and TLR within the first year, whereas late restenosis and late TLR beyond 1 year has been more common with DES use than with BMS use.^9,10^

Fully bioabsorbable drug-eluting stents have been developed as a new device that could potentially suppress the late adverse events related to the permanent presence of polymer and metallic devices. Very limited experiences of the fully bioabsorbable vascular scaffolds (Abbott Vascular) have demonstrated promising midterm clinical results, suggesting late luminal enlargement and restoration of vasomotion at the index stented site.^11–13^ However, long-term outcomes of the bioabsorbable drug-eluting stents are currently unknown. Very long-term outcomes in patients who were free from early restenosis after BA compared with after BMS implantation could provide some insight into whether fully bioabsorbable drug-eluting stents could prevent late adverse events related to permanent metal prosthesis. Therefore, we sought to investigate the very long-term outcomes of patients who had undergone successful BA in comparison with the previously reported outcomes of patients with successful BMS implantation.^5^

## Methods

### Study Population

**Figure 1.**
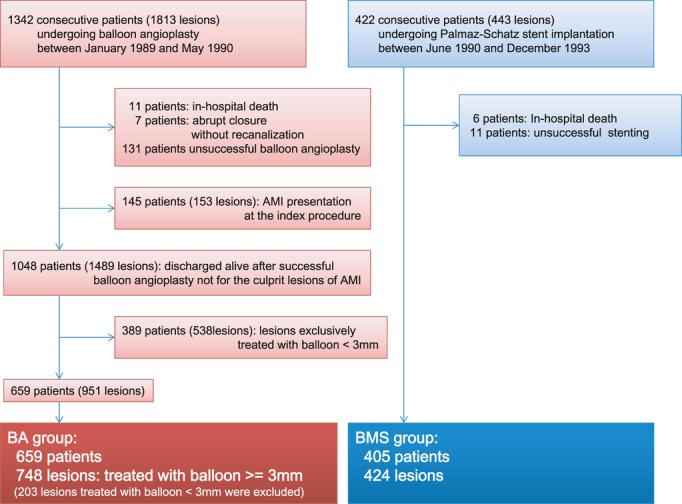
Study flow chart of patients in the BA group and the BMS group. AMI indicates acute myocardial infarction; BMS, bare-metal stents; BA, balloon angioplasty.

A successful procedure for both BA and BMS implantation was defined as dilatation of the target lesion with a final-diameter stenosis <50% either with or without non-flow-limiting dissections. The study population and very long-term outcomes of the BMS group in the current study were previously reported.^4,5,14^ In brief, percutaneous coronary interventional procedures including both BA and coronary artery stenting were attempted in 6059 patients (7466 lesions) in Kokura Memorial Hospital from June 1990 through December 1993. Of those, the BMS group included 405 patients (424 lesions) who were discharged alive after successful Palmaz-Schatz stent placement in the native coronary arteries (Figure 1).

To enroll those BA patients who were as comparable as possible with patients in the BMS group, we screened a total of 1342 consecutive patients (1813 lesions) who had undergone BA from January 1989 through May 1990, just before the recruitment period of the BMS patients. Of those, 149 patients were excluded from the current analysis: 11 patients who died in the hospital, 7 patients with abrupt closure without recanalization, and 131 patients with a final-diameter stenosis >50%. Because stents were not used in the culprit lesions for acute myocardial infarction in this period, we excluded 145 patients (153 lesions) who presented with acute myocardial infarction at the time of index balloon angioplasty procedure. To eliminate small vessels unsuitable for Palmaz-Schatz stent placement, we excluded 389 patients (538 lesions) who were exclusively treated by angioplasty with balloon diameter <3.0 mm, consistent with our previous report.^15^ We also excluded 203 lesions dilated using balloon diameter <3.0 mm in patients who had at least 1 lesion treated with angioplasty with balloon diameter ≥3.0 mm. Finally, the study population for the BA group consisted of 659 patients (748 lesions) who underwent successful balloon angioplasty using final balloon ≥3.0 mm excluding the culprit lesion of acute myocardial infarction (Figure 1).

All the study patients gave written informed consent for the procedure and the follow-up protocol, which was approved by the institutional review board.

### Clinical Follow-Up

Clinical information was obtained either from a review of the hospital record or by telephone contacts with the patients, the family members, or the primary care physicians. Death was regarded as cardiac in origin unless obvious noncardiac causes could be identified. Myocardial infarction (MI) was defined as an increase in serum creatine kinase activity to more than twice the normal value in association with new pathological Q waves. Target lesion revascularization (TLR) was defined as either target lesion PCI (TL-PCI) or coronary artery bypass grafting (CABG) for restenosis of the target lesion (TL-CABG). Target lesion was defined as lesions including the edge segments as well as the ostium of the side branches.

**Figure 2.**
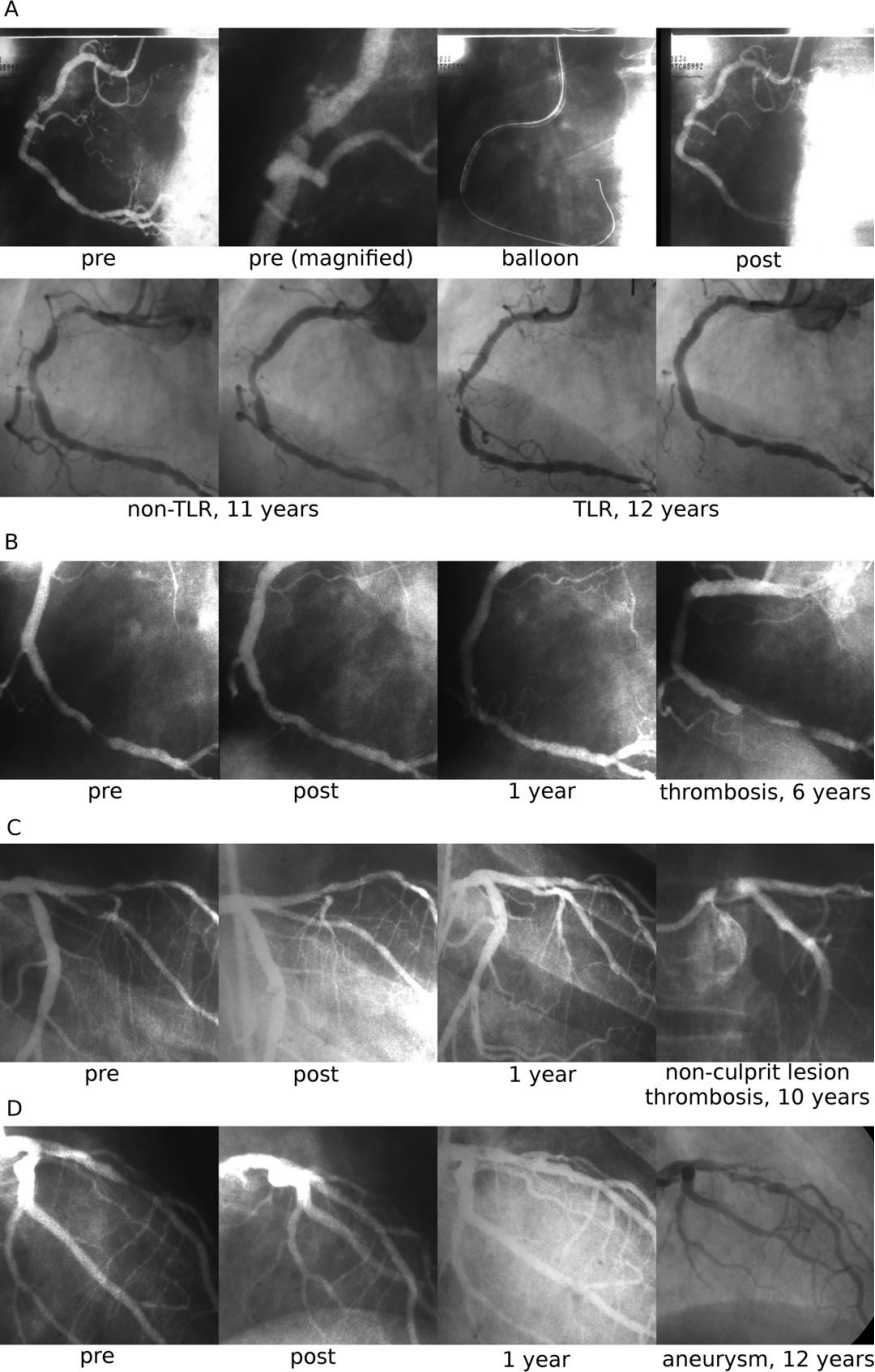
Representative cases of balloon angioplasty. A, Angiograms of a patient with non-TLR and TLR; B, angiograms of a patient with thrombosis at the target lesion; C, angiograms of a patient with a myocardial infarction related to adjacent nontarget lesion; D, angiograms of a patient with late aneurysm formation that was not evident at 1 year. TLR indicates target lesion revascularization.

In patients with target vessel revascularization (TVR), either TLR or non-TLR TVR was discriminated by reviewing digital images of dilated balloons or implanted stents at the index procedure side by side with the angiogram at the time of TVR, as shown in Figure 2A. Late TLR was defined as the first TLR performed beyond 4 years after the index procedure. The specific duration of 4 years was chosen because the cumulative incidence of TLR reached a plateau from 14 months to 4 years, and progressive increase in late TLR was observed beyond 4 years at the rate of 1.6% per year in our previous report in patients with BMS implantation.^5^ Data on angina status according to Canadian Cardiovascular Society (CCS) classification were collected at the time of TLR.^16^

### Angiographic Follow-Up

The early angiographic studies, at 6 months, were performed according to the institutional protocol for both BA and BMS groups. All patients were encouraged to undergo follow-up angiography at 3 and 6 months as the institutional protocol, whereas the follow-up angiographic studies at 1 and 3 years were dictated by the study protocol only for the initial 143 patients (35.3%) in the BMS group. Patients who underwent unscheduled follow-up angiography for clinical reasons were also included for serial angiographic analysis. The angiographic studies beyond 4 years were conducted according to clinical indications. Early, long-term, and very long-term studies were defined as those performed between 4 and 14 months, between 4 and 10 years, and beyond 10 years, respectively.^5^ Follow-up angiography at the time of TLR was included for quantitative angiographic analysis (QCA) in the serial angiographic study, whereas follow-up angiography in patients with prior TLR was excluded from the analysis. QCA was performed with the Cardiovascular Angiography Analysis System II, as described previously.^5^

### Statistical Analysis

Categorical variables and ordinal variables are expressed as numbers and percentages. Frequency analysis was performed with the χ^2^ test. Continuous variables are expressed as mean±SD and/or (range) or median (range and/or interquartile range [IQR]) and were compared with the unpaired *t* test or the Wilcoxon rank sum test, depending on their distributions. Paired variables obtained from the serial quantitative angiographic analyses were compared by the paired *t* test. Cumulative incidences were calculated by the Kaplan–Meier method and were compared using the log-rank test. Cumulative incidence estimates of TLR accounting for competing risk of death were also calculated.^17^ To adjust for differences in clinical and angiographic characteristics between the BA and BMS groups, the multivariable Cox proportional hazard model was used for comparisons of outcomes between the 2 groups. We confirmed that proportional hazard assumption was acceptable on the plots of log (time) versus log (−log [survival]) for all-cause death. Regarding TLR, the proportionality assumption was also acceptable for the long-term observation, although the proportionality assumption was not met in the first year after the index procedure. We included all the clinical variables listed in Table 1 in the multivariable Cox proportional hazard models.

**Table 1.** Baseline Patient, Lesion, and Procedural Characteristics

	BA	BMS	*P*
Patient characteristics			

Number of patients	659	405	

Age, years (range)	63±10 (36 to 86)	64±9 (34 to 84)	0.03

Male sex, n (%)	515 (78)	320 (79)	0.74

Extent of coronary artery disease, n (%)			0.17

Single vessel disease	320 (49)	179 (44)	
	
Multivessel disease	339 (51)	226 (56)	

Prior coronary artery bypass grafting, n (%)	13 (2.0)	26 (6.4)	<0.001

Prior myocardial infarction, n (%)	370 (56)	221 (55)	0.62

Left ventricular dysfunction (LVEF <40%), n (%)	37 (5.6)	47 (12)	<0.001

Class 3 or 4 angina, n (%)	174 (26)	192 (48)	<0.001

Hypertension, n (%)	298 (45)	180 (44)	0.80

Hypercholesterolemia, n (%)	198 (30)	130 (32)	0.48

Diabetes mellitus, n (%)	153 (23)	117 (29)	0.04

Renal failure (serum creatinine >1.3 mg/dL), n (%)	96 (15)	51 (13)	0.38

Smokers, n (%)	212 (32)	105 (26)	0.03

Lesion characteristics			

Number of lesions	748	424	

Number of lesions per patients	1.14	1.04	

Lesion location, n (%)			<0.001

Left anterior descending coronary artery	390 (52)	181 (43)	
	
Right coronary artery	248 (33)	183 (43)	
	
Left circumflex coronary artery	108 (14)	46 (11)	
	
Left main coronary artery	2 (0.3)	14 (3.3)	

Restenotic lesion, n (%)	113 (15)	177 (42)	<0.001

Minimum lumen diameter before procedure, mm	0.93±0.41	0.98±0.38	<0.001

Minimum lumen diameter after procedure, mm	1.88±0.37	2.61±0.42	<0.001

Reference diameter before procedure, mm	2.90±0.56	3.16±0.55	<0.001

Lesion length, mm	12.4±5.8	10.4±5.6	<0.001

Final balloon size, mm	3.06±0.2	3.53±0.4	<0.001

BA indicates balloon angioplasty; BMS, bare metal stent; LVEF, left ventricular ejection fraction.

Landmark analysis at 4 years was performed to evaluate the difference in the cumulative incidence of late TLR between the BA and BMS groups. Patients who were free from death and TLR without loss to follow-up at 4 years were included for further survival analysis. Very long-term outcomes in patients who were free from early restenosis after BA compared with those after BMS could provide some insight into whether fully bioabsorbable drug-eluting stents could prevent late adverse events related to permanent metal prosthesis. However, the initial report of the bioabsorbable vascular scaffolds (Abbott Vascular) demonstrated that percent diameter stenosis (%DS) at 6 months and 2 years was 27±14% and 27±11%, respectively, which seemed smaller than that in patients receiving BA.^11^ Because the difference in the degree of residual stenosis between BA and fully bioabsorbable drug-eluting stents could potentially influence late clinical outcomes, dichotomized analysis was also conducted in the BA group according to the %DS at the early follow-up angiography (small %DS group and large %DS group). In patients who had multiple treated lesions, average %DS was used for dichotomization. Cumulative incidence of late TLR was compared between the small and large %DS groups.

All statistical analyses were performed using JMP 9.03 and SAS 9.2 (SAS Institute, Cary, NC). All reported *P* values were 2-sided, and *P* values <0.05 were regarded as statistically significant.

## Results

### Baseline Characteristics

Patients who underwent balloon angioplasty and those with stenting differed significantly in clinical and angiographic characteristics. Patients in the BMS group more often had high-risk clinical features such as prior coronary artery bypass grafting, left ventricular dysfunction, and class 3 or 4 angina, whereas patients in the BA group had more complex lesion characteristics such as smaller vessel diameter, longer lesion length, and greater number of lesions treated per patient (Table 1).

### Clinical Follow-Up Outcomes

Late clinical follow-up information in the BA and BMS groups was obtained in 610 (93%) and 398 (98%) patients at 5 years, 552 (84%) and 340 (84%) patients at 10 years, and 529 (80%) and 327 (81%) patients at 15 years, respectively (*P*=0.85). The median follow-up interval of the 338 survivors in the BA group (17.6 years, IQR 9.2 to 21.2 years) was significantly longer compared with 228 survivors in the BMS group (15.9 years, IQR 9.6 to 17.4 years) (*P*<0.001).

**Figure 3.**
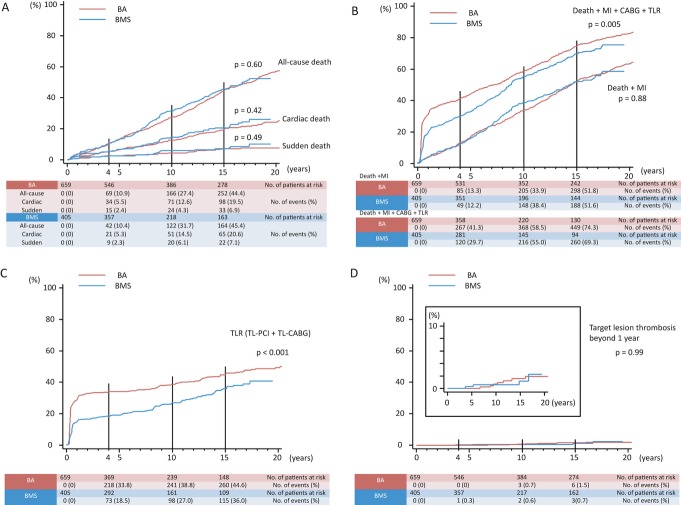
A, Kaplan–Meier curves for all-cause death, cardiac death, and sudden death; B, Kaplan–Meier curves for the composite of death, myocardial infarction, coronary artery bypass grafting, and TLR and for the composite of death and myocardial infarction; C, Kaplan–Meier curves for TLR during overall follow-up; D, Kaplan–Meier curves for target lesion thrombosis beyond 1 year. BA indicates balloon angioplasty; BMS, bare metal stent; MI, myocardial infarction; TLR, target lesion revascularization; PCI, percutaneous coronary intervention; and CABG, coronary artery bypass grafting.

The cumulative incidence of all-cause death was similar between the BA and BMS groups (44.4% versus 45.4%, *P*=0.60) (Table 2, Figure 3A). The distribution of the causes of death was similar between the 2 groups, and the proportion of cardiac death was similar (around 40%) in both groups (Table 3). After adjusting for potential confounders, the risk of the BMS group relative to the BA group for all-cause death was not significant (adjusted HR 0.95 [95% CI 0.78 to 1.16], *P*=0.64) (Table 4). The cumulative incidence of the composite of death or MI at 15 years (52.0% versus 51.6%, log-rank *P*=0.88) was also similar, whereas the cumulative incidence of the composite of death, MI, CABG, or TL-PCI at 15 years was significantly higher in the BA group than in the BMS group (74.3% versus 69.3%, log-rank *P*=0.005) (Figure 3B).

**Table 2.** Cumulative Incidences of Clinical Events

	14 Months		4 Years		10 Years		15 Years	
				
	BA	BMS	BA	BMS	BA	BMS	BA	BMS
All-cause death	26 (4.0)	19 (4.7)	69 (10.9)	42 (10.4)	166 (27.4)	122 (31.7)	252 (44.4)	164 (45.4)

Cardiac death	15 (2.3)	13 (3.2)	34 (5.5)	21 (5.3)	71 (12.6)	51 (14.5)	98 (19.5)	65 (20.6)

Sudden death	6 (0.9)	7 (1.8)	15 (2.4)	9 (2.3)	24 (4.3)	20 (6.1)	33 (6.9)	22 (7.1)

Vascular death	4 (0.6)	5 (1.3)	15 (2.5)	10 (2.6)	29 (5.3)	25 (7.3)	42 (9.0)	30 (9.8)

Noncardiovascular death	7 (1.1)	1 (0.3)	20 (3.3)	11 (2.9)	66 (12.4)	46 (13.8)	112 (24.0)	69 (23.8)

MI	5 (0.8)	2 (0.5)	24 (4.1)	9 (2.4)	65 (12.3)	36 (10.9)	85 (18.1)	43 (14.8)

CABG	13 (2.0)	8 (2.0)	17 (2.7)	22 (5.8)	33 (6.0)	35 (10.0)	56 (12.9)	46 (15.8)

TLR	203 (31.5)	64 (16.1)	217 (33.8)	73 (18.5)	240 (38.8)	98 (27.0)	259 (44.6)	115 (36.0)

TLR taking competing risk of death into account	203 (31.2)	64 (15.8)	217 (33.4)	73 (18.0)	240 (37.3)	98 (24.7)	259 (41.1)	115 (30.2)

Any coronary revascularization	251 (38.9)	140 (35.2)	293 (45.9)	175 (44.7)	355 (58.4)	228 (61.6)	386 (67.6)	248 (71.8)

Data are expressed as number of patients with events (cumulative incidence [%]). BA indicates balloon angioplasty; BMS, bare metal stent; MI, myocardial infarction; CABG, coronary artery bypass grafting; TLR, target lesion revascularization.

**Table 3.** Causes of Death

	BA (n=321)	BMS (n=177)
Cardiac death, n (%)	117 (36)	72 (41)

Sudden death	35 (11)	25 (14)

Congestive heart failure	29 (9.0)	18 (10)

Acute myocardial infarction	23 (7.2)	11 (6.2)

Postoperative death (CABG)	4 (1.2)	3 (1.7)

Postoperative death (PCI)	2 (0.6)	0 (0)

Unknown	24 (7.5)	15 (8.5)

Vascular death, n (%)	61 (19)	30 (17)

Stroke	36 (11)	12 (6.8)

Chronic renal failure	8 (2.5)	10 (5.6)

Aortic aneurysm	15 (4.7)	6 (3.4)

Pulmonary embolism	1 (0.3)	1 (0.6)

Systemic embolism	0 (0.0)	1 (0.6)

Cholesterol crystal embolism	1 (0.3)	0 (0.0)

Noncardiovascular death, n (%)	143 (45)	75 (42)

BA indicates balloon angioplasty; BMS, bare metal stent; CABG, coronary artery bypass grafting; PCI, percutaneous coronary intervention.

**Table 4.** Univariate and Multivariate Correlates for All-Cause Death, TLR During Overall Follow-Up and Late TLR (>4 Years)

	Univariate		Multivariable	
		
Variables	HR (95% CI)	*P*	HR (95% CI)	*P*
***All-cause death***				

BMS group	1.05 (0.87 to 1.27)	0.60	0.95 (0.78 to 1.16)	0.64

Age	1.08 (1.07 to 1.10)	<0.001	1.08 (1.07 to 1.10)	<0.001

Male sex	0.85 (0.70 to 1.06)	0.16	1.13 (0.90 to 1.42)	0.28

Single-vessel disease	0.72 (0.60 to 0.86)	<0.001	0.98 (0.81 to 1.19)	0.85

Prior coronary artery bypass grafting	1.10 (0.69 to 1.71)	0.69	0.98 (0.58 to 1.55)	0.92

Prior myocardial infarction	1.20 (1.01 to 1.44)	0.04	1.12 (0.93 to 1.36)	0.24

Left ventricular dysfunction (LVEF <40%)	2.22 (1.65 to 2.91)	<0.001	1.85 (1.35 to 2.50)	<0.001

Class 3 or 4 angina	1.04 (0.86 to 1.25)	0.71	0.95 (0.78 to 1.16)	0.62

Hypertension	1.15 (0.96 to 1.37)	0.13	1.08 (0.90 to 1.30)	0.38

Hypercholesterolemia	0.97 (0.79 to 1.16)	0.65	1.07 (0.88 to 1.30)	0.49

Diabetes mellitus	1.50 (1.23 to 1.81)	<0.001	1.53 (1.26 to 1.87)	<0.001

Renal failure (serum creatinine >1.3 mg/dL)	1.44 (1.14 to 1.80)	0.003	1.15 (0.90 to 1.45)	0.27

Smokers	0.84 (0.69 to 1.02)	0.08	1.11 (0.90 to 1.36)	0.35

***TLR during overall follow-up***				

BMS group	0.64 (0.51 to 0.79)	<0.001	0.62 (0.50 to 0.78)	<0.001

Age	0.99 (0.98 to 1.00)	0.007	0.99 (0.98 to 1.00)	0.03

Male sex	1.48 (1.14 to 1.96)	0.003	1.42 (1.07 to 1.90)	0.01

Single-vessel disease	0.88 (0.72 to 1.07)	0.20	0.88 (0.71 to 1.09)	0.23

Prior coronary artery bypass grafting	1.26 (0.74 to 1.98)	0.37	1.41 (0.82 to 2.26)	0.20

Prior myocardial infarction	1.07 (0.88 to 1.31)	0.51	0.99 (0.80 to 1.22)	0.93

Left ventricular dysfunction (LVEF <40%)	0.92 (0.60 to 1.34)	0.66	0.96 (0.62 to 1.43)	0.85

Class 3 or 4 angina	0.88 (0.71 to 1.09)	0.24	0.96 (0.77 to 1.19)	0.70

Hypertension	1.04 (0.85 to 1.27)	0.69	1.03 (0.84 to 1.26)	0.80

Hypercholesterolemia	0.86 (0.69 to 1.07)	0.17	0.87 (0.69 to 1.08)	0.20

Diabetes mellitus	1.34 (1.07 to 1.66)	0.01	1.39 (1.10 to 1.74)	0.005

Renal failure (serum creatinine >1.3 mg/dL)	1.19 (0.89 to 1.56)	0.23	1.14 (0.85 to 1.51)	0.38

Smokers	1.11 (0.90 to 1.37)	0.33	0.99 (0.79 to 1.24)	0.94

***Late TLR (>4 years)***				

BMS group	1.33 (0.89 to 1.97)	0.17	1.42 (0.92 to 2.18)	0.11

Age	0.96 (0.93 to 0.98)	<0.001	0.95 (0.93 to 0.97)	<0.001

Male sex	1.35 (0.82 to 2.35)	0.25	1.10 (0.64 to 1.98)	0.73

Single-vessel disease	0.96 (0.65 to 1.42)	0.84	0.84 (0.55 to 1.30)	0.43

Prior coronary artery bypass grafting	1.05 (0.26 to 2.78)	0.94	0.92 (0.22 to 2.62)	0.90

Prior myocardial infarction	0.88 (0.60 to 1.30)	0.53	0.77 (0.51 to 1.16)	0.21

Left ventricular dysfunction (LVEF <40%)	0.52 (0.13 to 1.39)	0.22	0.58 (0.14 to 1.60)	0.32

Class 3 or 4 angina	1.14 (0.76 to 1.70)	0.52	0.99 (0.64 to 1.50)	0.96

Hypertension	0.80 (0.52 to 1.19)	0.27	0.73 (0.48 to 1.11)	0.14

Hypercholesterolemia	0.98 (0.63 to 1.47)	0.92	0.88 (0.56 to 1.35)	0.58

Diabetes mellitus	1.43 (0.89 to 2.22)	0.13	1.39 (0.82 to 2.21)	0.18

Renal failure (serum creatinine >1.3 mg/dL)	1.01 (0.52 to 1.76)	0.99	1.26 (0.64 to 2.27)	0.49

Smokers	1.00 (0.65 to 1.51)	1.00	0.87 (0.55 to 1.36)	0.55

TLR indicates target lesion revascularization; BMS, bare metal stent; LVEF, left ventricular ejection fraction.

**Figure 4.**
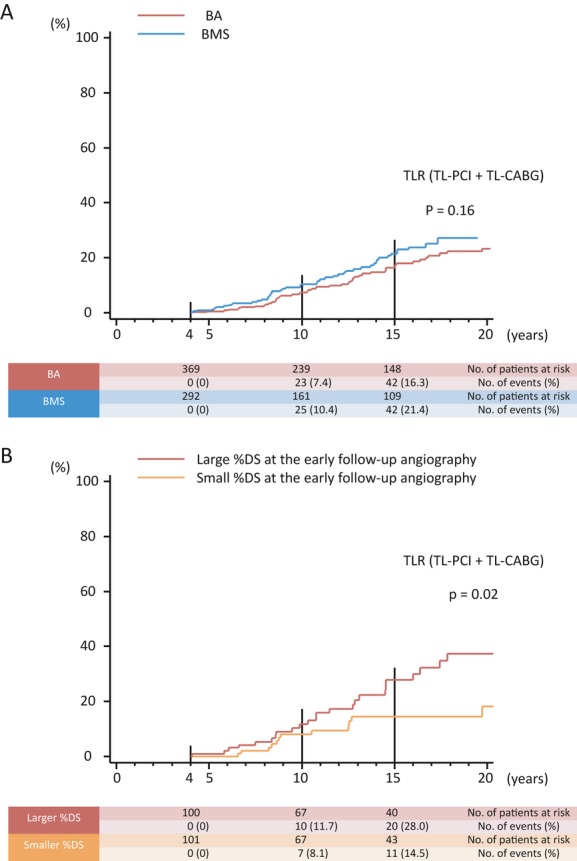
A, Landmark analysis for late TLR comparing the BA group with the BMS group; B, landmark analysis for late TLR in dichotomized BA patients according to %DS at early follow-up angiography. BA indicates balloon angioplasty; BMS, bare metal stent; MI, myocardial infarction; TLR, target lesion revascularization; PCI, percutaneous coronary intervention; CABG, coronary artery bypass grafting; and %DS, percent diameter stenosis.

The cumulative incidence of TLR during the overall follow-up period was significantly higher in the BA group than in the BMS group (44.6% versus 36.0% at 15 years, log-rank *P*<0.001; adjusted HR 0.62 [95% CI 0.50 to 0.78], *P*<0.001) (Figure 3C). The difference in TLR rates was most marked within the first year after the index procedure. A total of 369 patients in the BA group and 292 patients in the BMS group were included in the landmark analysis at 4 years. Late TLR (>4 years) through 15 years was performed in 42 patients (21.4%) in the BMS group, compared with 42 patients (16.3%) in the BA group (log-rank *P*=0.16; adjusted HR 1.42 [95% CI 0.92 to 2.18], *P*=0.11) (Figure 4A).

Symptomatic status at the time of late TLR in the BA and BMS groups was generally comparable except for the slightly greater prevalence of acute myocardial infarction presentation in the BA group (*P*=0.38) (Table 5). Among those 10 patients who underwent late TLR for acute myocardial infarction in the BA group, TLR was conducted due to target lesion thrombosis in 7 patients, MI related to adjacent nontarget lesion in 2 patients, and nontarget vessel MI treated with multivessel CABG (Figure 2B and 2C). More than 1 year after the index procedure, target lesion thrombosis without prior TLR occurred in 7 patients in the BA group, compared with 4 patients in the BMS group (1.5% versus 0.7%, respectively, log-rank *P*=0.99) (Figure 3D).

**Table 5.** Symptomatic Status at Time of Late TLR (>4 Years)

Group	BA (n=54)	BMS (n=47)
Acute myocardial infarction	10 (19%)	4 (8.5%)

Class 3 or 4 angina	13 (24%)	15 (32%)

Class 1 or 2 angina	13 (24%)	11 (23%)

Asymptomatic	14 (26%)	17 (36%)

Silent myocardial ischemia detected by noninvasive tests	9 (17%)	8 (17%)

Restenosis at time of cardiovascular surgery or follow-up angiography for non-TLR	3 (5.6%)	1 (2.1%)

Data not available	2 (3.7%)	8 (17%)

Symptomatic status not available	4 (7.4%)	0 (0.0%)

BA indicates balloon angioplasty; BMS, bare metal stent; TLR, target lesion revascularization; and non-TLR, nontarget lesion revascularization.

Early TLR (≤4 years) was performed in 218 patients (33.8%) in the BA group and in 73 patients (18.5%) in the BMS group. Among those patients who were free from death or TLR without loss to follow-up at 4 years, 201 patients (54.5%, 218 lesions) in the BA group and 279 patients (95.5%, 286 lesions) in the BMS group underwent early follow-up angiography. Minimum lumen diameter (MLD) at the early follow-up angiography was significantly smaller in the BA group (1.93±0.43 mm) than in the BMS group (2.10±0.50 mm) (*P*<0.001), whereas %DS was significantly larger in the BA group (35.7±11.7%) than in the BMS group (33.4±13.9%) (*P*=0.049).

According to the median %DS (37% [IQR 27.5% to 44%]) at the early follow-up angiography, patients in the BA group were dichotomized into the 2 groups (small %DS group and large %DS group). The average %DS and MLD in the small and large %DS groups were 26.7±7.1% versus 44.7±6.3% and 2.16±0.37 versus 1.70±0.29 mm, respectively. The cumulative incidence of late TLR was significantly lower in patients with small %DS at early follow-up angiography compared with those with large %DS (14.5% versus 28.0% at 15 years, log-rank *P*=0.02; Figure 4B).

### Angiographic Follow-Up Outcomes

In the BA and BMS groups, early, long-term, and very long-term angiographic follow-up without intercurrent TLR were performed in 344 patients (52%, 381 lesions) versus 394 patients (95%, 412 lesions), 87 patients (22%, 94 lesions) versus 168 patients (57%, 175 lesions), and 62 patients (20%, 65 lesions) versus 74 patients (35%, 76 lesions), respectively. Median intervals from the index coronary intervention to the follow-up angiographic studies in the BA and BMS groups were 6.8 (IQR 4.3 to 12.0) months versus 6.2 (IQR 6.0 to 6.8) months (*P*<0.001) for the early study, 87.5 (IQR 68.0 to 103.5) months versus 77.9 (IQR 65.0 to 95.4) months (*P*=0.08) for the very long-term study, and 163.1 (IQR 145.4 to 184.1) months versus 166.2 (IQR 144.3 to 184.3) months (*P*=0.77), respectively.

**Figure 5.**
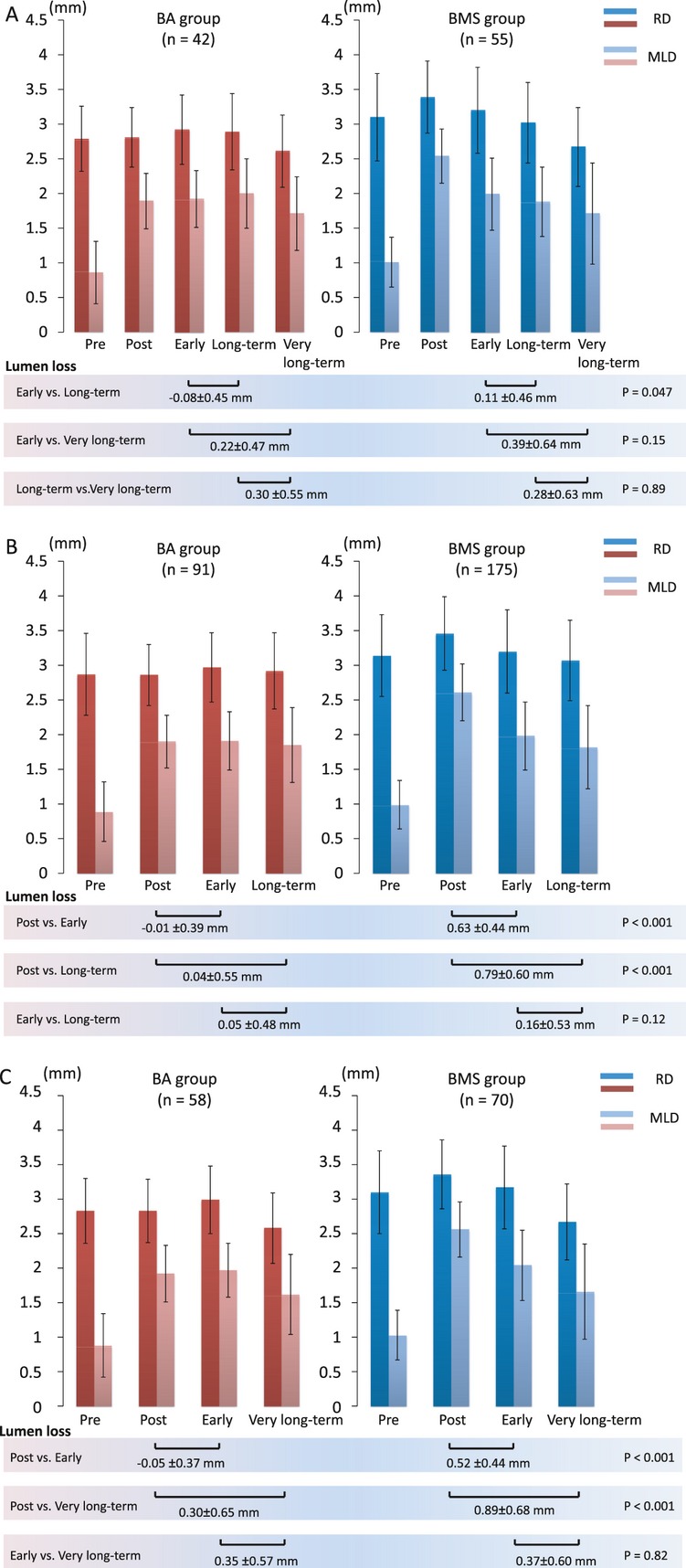
Serial quantitative coronary angiographic analysis in the BA and in BMS groups. A, Patients with complete serial analysis; B, patients with paired angiograms at early and long-term follow-up; C, patients with paired angiograms at early and very long-term follow-up. BA indicates balloon angioplasty; BMS, bare metal stent; RD, reference diameter; and MLD, minimal luminal diameter.

Complete serial angiographic studies without intercurrent TLR were performed in 42 lesions in the BA group and 55 lesions in the BMS group. Late lumen loss from the early to the long-term follow-up angiography was significantly smaller in the BA group (−0.08±0.45 mm) compared with the BMS group (0.11±0.46 mm) (*P*=0.047), whereas late loss from the long-term to the very long-term follow-up angiography was similar between the 2 groups (0.30±0.55 versus 0.28±0.63 mm) (*P*=0.89) (Figure 5A).

In 91 BA patients who underwent both early and long-term follow-up angiography without intercurrent TLR, MLD remained stable between the early (1.91±0.42 mm) and the long-term (1.85±0.54 mm) follow-up angiography (*P*=0.26). Lumen loss from the early to the long-term follow-up angiography was not statistically different between the BA group (n=91, 0.05±0.48 mm) and the BMS group (n=175, 0.16±0.53 mm) (*P*=0.12) (Figure 5B. In 58 BA patients who underwent both early and very long-term follow-up angiography without intercurrent TLR, MLD decreased from 1.97±0.39 to 1.62±0.58 mm (*P*<0.001). Lumen loss from the early to the very long-term follow-up angiography was not statistically different between the BA group (n=58, 0.35±0.57 mm) and the BMS group (n=70, 0.37±0.60 mm) (*P*=0.82) (Figure 5C).

Regarding abnormal angiographic findings in the BA group, late aneurysm formation was newly observed in 2 patients at very late follow-up angiography (Figure 2D).

## Discussion

The main findings of the current study were as follows: (1) the cumulative incidence of late TLR beyond 4 years after BA tended to be lower than that after BMS implantation; (2) patients with small %DS at the early follow-up angiography after BA were associated with lower incidence of late TLR than patients with large %DS; (3) late lumen loss from the early to the long-term follow-up angiography was significantly smaller after BA than after BMS implantation; (4) cumulative incidences of all-cause death, cardiac death, sudden death, and late target lesion thrombosis were similar between the BA and BMS groups.

The current study reports the longest clinical and angiographic follow-up outcomes comparing BA and BMS stenting. We previously reported that late TLR beyond 4 years after BMS implantation had not occurred infrequently (1.6% per year) after an apparent plateau phase between 1 and 4 years.^5^ In the current analysis, the cumulative incidence of late TLR after BA (1.0% per year) tended to be lower than that after BMS implantation (1.6% per year). Although the difference did not reach statistical significance, we should consider several differences in lesion and procedural characteristics between BA and BMS implantation. The BA group included high-risk angiographic characteristics such as smaller vessel diameter, longer lesion length, and greater number of lesions treated per patient, and the majority of lesions in the BA group were dilated, with balloon size of 3.0 mm, compared with a final balloon size of 3.53±0.4 mm in the BMS group. The proportion of dilated segment (ie, risk segment for TLR) in the entire coronary tree was certainly greater with BA than with BMS implantation. Furthermore, degree of residual stenosis at the early angiographic follow-up study was greater with BA than with BMS implantation.

Regarding serial changes in minimal luminal diameter after BA, Hatrick et al reported slow late luminal loss from a late follow-up study at 4.5 years to a very late follow-up study at 12 years, which were almost equivalent to the luminal gain by early regression from an early follow-up study at 7 months to a late follow-up study at 4.5 years.^18^ Yamada et al also reported late luminal renarrowing over 7 years after a regression phase up to 3 to 5 years after BA.^19^ The angiographic outcomes of the current study were in line with these observations. It is important to note that despite significant late luminal renarrowing observed during 15 years of follow-up after BA, late lumen loss from the early to the long-term follow-up angiography was significantly smaller in the BA group compared with the BMS group. Significantly smaller late lumen loss in long-term follow-up together with a trend for less late TLR beyond 4 years after BA compared with after BMS implantation might suggest that the permanent presence of a metallic prosthesis in the coronary artery was predisposing to more aggressive atherosclerotic progression.

It is intriguing that target lesion thrombosis beyond 1 year was demonstrated to occur after BA with a frequency similar to that after BMS implantation. The absence of permanent metal was not necessarily associated with freedom from target lesion thrombosis. Disruption of in-stent neoatherosclerosis has been suggested as one of the pathogenetic mechanisms of very late stent thrombosis of BMS.^7,20^ Because of the paucity of long-term histopathologic analysis in patients treated with BA, the mechanisms of late target lesion thrombosis are only speculative. It is not yet clarified whether late target lesion thrombosis is related to further expansion of the original atherosclerotic plaque or to neoatherosclerosis formation within neointimal tissue.

It also should be noted that the adjusted hazard ratios of the BMS group relative to the BA group for mortality and composite of death and MI were not significant despite a significantly lower risk for TLR. This observation was consistent with a meta-analysis of randomized controlled trials comparing DES with BMS, suggesting that the risk for all-cause mortality was similar between DES and BMS despite a significantly lower risk for TLR after DES implantation.^21^

Given the significantly smaller late lumen loss in long-term follow-up together with a trend for less late TLR beyond 4 years after BA compared with after BMS implantation, complete bioabsorption of the scaffold a few years after implantation might potentially reduce the late adverse events provoked by the permanent presence of polymer and metallic prostheses. The biggest difference between BA and fully bioabsorbable DES is the degree of stenosis after the initial restenosis phase, favoring fully bioabsorbable DES. From the 2-year outcomes of the ABSORB trial, mean %DS at 6 months was 27%, which was comparable to that observed in the BA patients with small %DS in the current study.^11^ Further reduction of the incidences of late adverse events might be expected with the use of fully bioabsorbable DES compared with BA, because a lesser degree of stenosis at the early follow-up study was associated with less late TLR in the current study.

The present study has several important limitations. First, many possible candidates for late TLR analysis were excluded from the 4-year landmark analysis, because approximately one third of the patients in the BA group underwent TLR within 4 years. As the long-term and very long-term angiographic follow-up analyses were conducted only in patients who were free from prior TLR, late lumen loss at the long-term and the very long-term angiographic follow-up could have been underestimated in patients with the BA group compared with in the BMS group. Second, the present study was not a randomized trial. Patients who underwent BMS implantation in the early period of coronary stenting were highly selected patients, and therefore clinical and angiographic characteristics of the BMS group were markedly different from those of the BA group. Third, although we made every effort to collect follow-up information, the long-term follow-up rate was far from complete. Furthermore, because of the relatively small numbers of patients, there was a possibility of type II error for the current analyses. Fourth, long-term and very long-term angiographic follow-up studies were not dictated by the protocol but were conducted on the basis of clinical needs, suggesting the presence of a selection bias toward evaluating more symptomatic patients. Furthermore, patients in the BMS group underwent nonclinically driven follow-up angiography more frequently than did those in the BA group, leading to higher rate of nonclinically driven TLR in the BMS group. Fifth, imaging analyses using intravascular ultrasound and optical coherence tomography were not performed. Finally, we would also note that a BMS over the moderate- and long term may not provide a template for what will happen to a DES with absorbable polymer, just as the long-term outcome of traditional balloon angioplasty may not provide a template for what will happen with totally bioabsorbable scaffolds, because our understanding of the processes involved with absorption of polymer embedded in the arterial wall is not clear.

## Conclusions

Compared with BMS implantation, BA was associated with a trend for less late TLR beyond 4 years and with significantly smaller late lumen loss from the early to the long-term follow-up angiography.
